# Psychometric properties of the EQ-5D-5L for aboriginal Australians: a multi-method study

**DOI:** 10.1186/s12955-021-01718-8

**Published:** 2021-03-10

**Authors:** Pedro Henrique Ribeiro Santiago, Dandara Haag, Davi Manzini Macedo, Gail Garvey, Megan Smith, Karen Canfell, Joanne Hedges, Lisa Jamieson

**Affiliations:** 1 Adelaide Dental School, The University of Adelaide, Adelaide, Australia; 2 School of Public Health, The University of Adelaide, Adelaide, Australia; 3 Menzies School of Health Research, Darwin, Australia; 4 Cancer Council of NSW, Sydney, Australia; 5 School of Public Health, University of Sydney, Sydney, Australia

## Abstract

**Introduction:**

In Australia, health-related quality of life (HRQoL) instruments have been adopted in national population surveys to inform policy decisions that affect the health of Aboriginal and Torres Strait Islanders. However, Western-developed HRQoL instruments should not be assumed to capture Indigenous conceptualization of health and well-being. In our study, following recommendations for cultural adaptation, an Indigenous Reference Group indicated the EQ-5D-5L as a potentially valid instrument to measure aspects of HRQoL and endorsed further psychometric evaluation. Thus, this study aimed to investigate the construct validity and reliability of the EQ-5D-5L in an Aboriginal Australian population.

**Methods:**

The EQ-5D-5L was applied in a sample of 1012 Aboriginal adults. Dimensionality was evaluated using Exploratory Graph Analysis. The Partial Credit Model was employed to evaluate item performance and adequacy of response categories. Area under the receiver operating characteristic curve (AUROC) was used to investigate discriminant validity regarding chronic pain, general health and experiences of discrimination.

**Results:**

The EQ-5D-5L comprised two dimensions, Physiological and Psychological, and reliability was adequate. Performance at an item level was excellent and the EQ-5D-5L individual items displayed good discriminant validity.

**Conclusions:**

The EQ-5D-5L is a suitable instrument to measure five specific aspects (Mobility, Self-Care, Usual activities, Pain/Discomfort, Anxiety/Depression) of Aboriginal and Torres Strait Islander HRQoL. A future research agenda comprises the investigation of other domains of Aboriginal and Torres Strait Islander HRQoL and potential expansions to the instrument.

## Introduction

It has been increasingly recognized by health policy makers, health practitioners and many population groups that biological parameters and clinical measures of disease are insufficient indicators of health status [1–4]. A growing body of evidence has documented the importance of subjective experiences and interpretation of health and illness on individuals’ quality of life [5]. This idea is central to the concept of Health-Related Quality of Life (HRQoL), which encompasses individuals’ evaluations of physical, psychological, and social well-being associated with their health state. Health-related quality of life assessments are useful in the fields of research, clinical practice and policy making. From a research perspective, these instruments can be adopted to assess HRQoL in clinical and epidemiological studies in order to assess the impacts associated with health conditions on individual’s well-being, as well as outcomes of healthcare interventions. In terms of policy implications, HRQoL assessments are useful for surveillance, as they support the development of evidence-based public health strategies, guiding the allocation of scarce resources. When adopted in healthcare settings, HRQoL measurements are a useful communication tool for identifying and prioritizing patient problems and preferences.

Although the importance of such measures is well established, a longstanding question remains on the applicability of such instruments among Indigenous populations [6]. This challenge arises from the different frames of reference in conceptualising health among Indigenous populations and Western societies, and is intensified by the narrow focus of the multiple HRQoL measures in biological aspects of health. Researching the field indicates that, while HRQoL instruments claim to capture subjective perceptions related to health, multiple HRQoL tools are actually generic health status measures [7, 8].

In Australia, the National Aboriginal Community Controlled Health Organisation (NACCHO) defines health and wellbeing as:

“…. not just the physical well-being of an individual but the social, emotional and cultural well-being of the whole Community in which each individual is able to achieve their full potential as a human being thereby bringing about the total well-being of their Community. It is a whole of life view and includes the cyclical concept of life-death-life.” [9]

This definition provides clear evidence that Aboriginal and/or Torres Strait Islander Australians have a concept of health and wellbeing that is distinct from the Western definition, encompassing elements such as community wellbeing and spiritual and cultural entities. Such conceptualization presents a holistic and multidimensional view of health and well-being. A comprehensive review of 95 articles by Butler, Anderson [6] identified nine domains of importance to the health and well-being of Indigenous Australians (autonomy, empowerment and recognition; family and community; culture, spirituality and identity; Country; basic needs; work, roles, and responsibilities; education; physical health; and mental health). Some of these domains are vaguely or not at all included in most Western HRQoL tools, such as spirituality, connection to Country and culture [6].

Theoretical criticisms for the use of HRQoL measures contribute further to the limitations in using such tools among Indigenous populations. One of the most important criticism relates to the strong emphasis on functional and role limitations placed by HRQoL measures, which may fail to assess the actual importance of these events on individuals’ lives. This questions to what extent the meaning of the impacts of diseases are assessed according to individuals’ personal beliefs, a central dimension for an Indigenous’ holistic view of health. Equally important is the consideration that since population perceptions of health and well-being vary across time and space, HRQoL tools are limited in the way they take these geographical and historical specificities into consideration. This is especially important for Aboriginal and Torres Strait Islander Australians, who are extremely diverse within themselves and who have been exposed to different geographical, political, economic and socio-historical determinants of health since colonisation [6].

Despite the abovementioned criticisms of the use of traditional HRQoL tools in the context of Indigenous health, these instruments have been largely uncritically adopted in national population surveys to inform policy decisions that affect the health of Aboriginal Australians. These include policy decisions such as the medication subsidy and health care performance evaluation (Department of Health: Canberra; 2016). To the best of our knowledge, there has been no HRQoL instrument developed that has been designed by and validated specifically for use among Australia’s Aboriginal and Torres Strait Islander population. While a more comprehensive and culturally appropriate HRQoL tool is not yet developed, it is of paramount importance that those being currently employed in research and policy development that affects Australian Aboriginal and Torres Strait Islander peoples provide some meaningful insights into the HRQoL of this population.

### Present research

In the current study, in partnership with Aboriginal groups in South Australia and following recommendations for the cultural adaptation of instruments [10], an Indigenous Reference Group was established and consulted regarding the face and content validity of a prominent HRQoL instrument, the EQ-5D-5L. The EQ-5D is a 5-item measure for describing and valuing health [11]. Over the decades, it became the most well-known and commonly applied instruments to measure HRQoL, being used both in clinical and non-clinical populations and translated to more than 160 languages [12, 13]. While the EQ-5D original instrument had only three response categories, the EQ-5D-5L was later developed to include five response categories, showing better discriminant capacity, increased reliability and reduced ceiling effects [13].

The EQ-5D-5L evaluates HRQoL through the five domains of mobility, self-care, usual activities, pain/discomfort, and anxiety/depression [13]. Upon examination of the instrument, the Indigenous Reference Group acknowledged the importance of capturing these HRQoL domains in Aboriginal and Torres Strait Islander populations, and in the absence of another suitable instrument being available endorsed its use. In addition to providing initial support for content and face validity, the Indigenous Reference Group advised that the EQ-5D-5L should undergo further psychometric evaluation; it is necessary, for instance, to investigate other aspects of construct validity, such as dimensionality and criterion validity.

In non-Indigenous cultures, a key feature of the EQ-5D-5L has been the derivation of “value sets” to weight responses by patients. The combination of the EQ-5D-5L five items each with five response categories (${5}^{5}$) describes 3125 unique health states. Individuals can then be asked, using preference-based methods such *time-trade off* or *standard gamble*, to indicate which health states they would prefer and value them between 0 (dead) and 1 (full health). These valuations are then attributed to each one of the 3125 unique health states, creating a continuum regarding which states are the least desirable and constituting a population-specific “value set” that can be used to calculate several quantities of interest, such as quality-adjusted life years (QALYs) (for an in-depth discussion about EQ-5D-5L preference-based valuation, please refer to Devlin and Krabbe [12]). While for non-Indigenous groups the derivation of “value sets” can be obtained through specified research guidelines [12], we followed recommendations from Young, Yang [14] that the “first stage of deriving a preference-based single-index measure for use in calculating quality-adjusted life years (QALYs) is to derive a health-state classification system that is *amenable* [emphasis added] to valuation using a preference-elicitation technique”. That is, prior to the calculation of “value sets” and subsequent assignment of values to health states, the first step of a HRQoL instrument validation is to ensure that the instrument correctly measures the health states intended to be measured. Only after construct validity is established, the instrument has been shown to provide valid measurement of health states of mobility, self-care, usual activities, ‘pain/discomfort’, and ‘anxiety/depression’ and is, consequently, amenable to preference-based techniques. Instrument validation prior to the application preference-based methods seems particularly important in Indigenous populations, in which the EQ-5D “logical inconsistencies suggests that the health state valuation instrument lacks construct validity” [15] and other Western-developed HRQoL measures were previously found to be “unsuitable for use” [16].

Hence, we performed the psychometric validation according the steps recommended by Young, Yang [14], consisting of: (1) identification of the instrument dimensionality; and (2) evaluation of the functioning of individual items (including the adequacy of response categories). To establish the instrument dimensionality, we investigated whether the EQ-5D-5L captured at least one or more dimensions of Aboriginal and Torres Strait Islanders’ HRQoL. After dimensionality was established, we examined whether the EQ-5D-5L individual items correctly measured mobility, self-care, usual activities, pain/discomfort, or anxiety/depression. Finally, we examined the instrument (3) criterion validity. To do so, we evaluated if EQ-5D-5L items scores could correctly identify Aboriginal and Torres Strait Islanders who had poor general health, were suffering from chronic pain or who experienced racial discrimination.

In summary, prior to the application of any preference-based techniques, this study examined whether the EQ-5D-5L could correctly measure in an Aboriginal population the health states intended to be measured (i.e. mobility, self-care, usual activities, pain/discomfort, and anxiety/depression). Similarly to many other countries and cultures in which the EQ-5D-5L has been officially validated [17], the validation (and possible adaption) of the EQ-5D-5L for Aboriginal and Torres Strait Islanders will inform whether this instrument can be used in future research and policymaking. The availability of a validated instrument is crucial to the measurement of HRQoL among Aboriginal Australians over the next years and to produce evidence that can be compared with population levels of HRQoL among other groups (such as Non-Aboriginal Australians). This evidence is absent at the moment and it is not clear how Aboriginal Australians stand in terms of HRQoL in comparison to other population groups. Moreover, once a validated HRQoL instrument is available, future studies can use preference-based techniques to derive utilities and calculate the health and financial impact of public policies on Aboriginal health. This evidence is likely to be used to inform and guide government policy in Australia over the next years. The aim of this study was to evaluate the construct validity and reliability of the EQ-5D-5L in an Aboriginal Australian population.

## Methods

### Participants and procedures

Data were from an overarching study which, as its primary outcome, aimed to investigate population estimates of oncogenic genotypes of oral HPV infection in Aboriginal and Torres Strait Islander populations. Inclusion criteria included being aged 18 + years and identifying as being Aboriginal and/or Torres Strait Islander. Recruitment strategies included: establishing service agreements with key Aboriginal community-controlled health organisations in South Australia, liaising with community champions, and encouraging word-of-mouth [18]. The study had six project staff who were led by a senior Indigenous project manager. The three non-Indigenous staff undertook extensive cultural competency training. A sample of 1012 Aboriginal adults was recruited at baseline and included several distinct language groups: Adnyamathanha, Akenta, Amarak, Bungandidj, Diyari, Erawirung, Kaurna, Kokatha Mula, Maralinga Tjarutja, Mirning, Mulbarapa, Narungga, Ngaanyatjarra, Ngadjuri, Ngarrindjeri, Nukunu, Parnkalla, Peramangk, Pitjantjatjara, Wirangu and Yankunjatjarra. All recruitment and data collection procedures were performed following the ethical standards laid down by the 1964 Declaration of Helsinki and its later amendments. Ethics approval was obtained from the University of Adelaide Human Research Ethics Committee (H-2016–246) and the Aboriginal Health Council of South Australia (04–17-729). All participants provided signed informed consent.

Missing responses of individual items ranged from 0.8% to 1.6%, meaning multiple imputation was not required [19]. Analyses were thus conducted with participants with complete questionnaire responses; that is, participants with responses to all EQ-5D-5L items. The final sample with complete questionnaire responses comprised 988 participants. These 988 participants were randomly and equally assigned into a test sample (n = 494) and validation sample (n = 494).

### Measures

#### EQ-5D-5L

The EQ-5D is a generic preference-based measure of health that evaluates health-related quality of life (HRQoL) according to five dimensions: Mobility, Self-Care, Usual Activities, Pain/Discomfort, and Anxiety/ Depression [11]. The EuroQoL Group recently introduced the EQ-5D-5L which expanded the original 3-category EQ-5D to include 5 categories [13]. In this study, the categories (response options) followed the format “no”, “slight,” “moderate”, “severe problems,” and “unable to”/ “extreme” for all dimensions.

#### Self-rated general health and chronic pain

General health was measured with a single-item question that asked: “Would you rate your general health as: (1) Excellent; (2) Very good; (3) Good; (4) Fair; (5) Poor”. While there are methodological challenges inherent to the validation of single-item questionnaires [20], single-item self-report measures of general health are considered important since they provide a holistic and integrated perception of Aboriginal health, including biological, psychosocial and social factors into the judgement [21]. For instance, Lavrencic, Mack [21] showed that single-item self-report measures of general health are associated with several health outcomes among Aboriginal Australians such as chronic diseases (e.g. arthritis and kidney problems) and perceived resilience. Following Lavrencic, Mack [21], in addition to the 5-point measure of general health (1 = Excellent, 2 = Very good, 3 = Good, 4 = Fair, 5 = Poor), we also created a new dichotomous variable so scores from “Excellent” to “Fair” indicated “good/fair health” and scores of “Poor” indicated “poor health”. The dichotomisation was done based on a ‘risk factor’ approach, aiming to identify the individuals with the highest risk (i.e. worst general health) [22]. This approach allows for the calculation of classification measures such as the area under the receiver operating characteristic curve (AUROC) [23], which can inform whether EQ-5D-5L scores correctly discriminate between individuals with good/fair and poor general health.

Chronic pain was measured with a single-item question that asked: “Do you now have significant pain that has lasted 6 months or more?” with response options “Yes” or “No”. Similar to previous research among Aboriginal Australians [24, 25], we evaluated self-report chronic pain instead of site-specific pain or clinical conditions causing pain. Previous research also showed that measures of self-report pain have been associated with criterion variables among Aboriginal Australians, such a high prevalence of multiple musculoskeletal conditions [25].

#### Experiences of racial discrimination

Experiences of racial discrimination were measured by 9 items that evaluate the frequency of racial discrimination in different settings (i.e. work, home, education, recreation, legal, medical, governmental, services and public) [26]. These items were based on the Measure of Indigenous Racism Experiences (MIRE), originally developed for Aboriginal and Torres Strait Islanders [27]. Items were rated on a 5-point scale ranging from “Strongly disagree” to “Strongly Agree”. For the experiences of racial discrimination, we also followed a ‘risk factor’ approach to distinguish between individual at high risk of experiences of racial discrimination and individuals with lower risk [22]. Hence, in addition to the MIRE total score (ranging from 9 to 45), we also created a new variable by dichotomising the MIRE total score according to the median, thus indicating participants with lower or higher frequency of experienced racial discrimination.

### Statistical analysis

#### Dimensionality

Exploratory Graph Analysis (EGA) [28] was used to investigate the EQ-5D-5L dimensionality in the test sample. EGA is a technique within the field of network psychometrics, a new scientific field dedicated to the study of psychological networks. Psychological networks are networks in which nodes represent items and edges represent the associations between items (e.g. partial correlations). In psychological networks, a *cluster* of items occurs when certain nodes are more strongly connected among each other compared to the rest of the network [29]. The aim of EGA is to identify these item clusters [30].

The first step of EGA is estimating a network model. The network model used in EGA was the Gaussian Graphical Model (GGM) [31], estimated with Least Absolute Shrinkage and Selection Operator (LASSO) [32] with turning parameter based on minimizing the Extended Bayesian Information Criteria (EBIC) [33]. After the network is estimated, EGA then employs a *walktrap algorithm* [34] to identify which items clustered in the psychological network. Since item clusters generate covariance patterns that are statistically equivalent to those produced by a latent variable [29], EGA can discover the instrument dimensionality by identifying the *number of item clusters*, in contrast to traditional factor analytical methods (e.g. Parallel Analysis) which identify the *number of latent variables* believed to connect the items. While an in-depth explanation of EGA is beyond the scope of this paper, accessible introductions to network psychometric and EGA can be found in Borsboom and Cramer [35] and Christensen, Golino [36], respectively.

Recent simulation studies showed that EGA performs as accurately as factor analytical procedures, such as the Automated Scree Test, Kaiser-Gutmman eigenvalue greater than 1 rule and Parallel Analysis, and outperforms them in large sample conditions. For example, in sample sizes of 500 respondents (similar to the test sample in our study), EGA discovered the correct number of factors in 81% of all simulated cases, while traditional procedures such as Kaiser-Gutmman eigenvalue greater than 1 rule discovered the correct number of factors only 70% of the time. Additionally, the EGA overall accuracy increases to 93% in samples of 5000 participants and its accuracy remained the highest among all methods to identify dimensionality independent of sample size [30].

The main output of EGA is a network plot in which nodes representing the five EQ-5D-5L items are coloured according to their identified dimensions. The network was plotted with the Fruchterman-Reingold algorithm [37], which arranges nodes more closely according to the strength of their associations (i.e. regularised partial correlations). One main reason we employed EGA over traditional methods is due to its graphical nature, providing an intuitive visual interpretation [30] of the associations established between the EQ-5D-5L items. Considering that EGA-identified dimensions are subject to sampling variation, we employed 2500 bootstrap samples to evaluate the stability of the identified dimensions and to ensure robustness of the results [38]. The analysis was conducted with R software [39] and the R package *EGAnet* [40].

#### Model fit

After the dimensional structure was identified by EGA in the *test* sample, we evaluated it with Confirmatory Factor Analysis (CFA) in the *validation* sample. The dimensional structure was confirmed in a different sample (that is, in the validation sample) to avoid overfitting [41] due to capitalization on sampling variation [42]. We compared the dimensional structure identified by EGA with the 1-dimensional model, which assumes that all five EQ-5D-5L items constitute a single dimension. The 1-dimensional model is the most parsimonious and, if a single dimension cannot be rejected, there is no reason to evaluate more complex structures [43]. CFA models were estimated with weighted least squares with a mean- and variance-adjusted (WLSMV) test statistic [44]. To evaluate model fit, the scaled χ2, scaled CFI and scaled RMSEA were used. Values of CFI $\ge$ 0.96 and RMSEA ≤ 0.05 indicate good model fit [45], while RMSEA ≤ 0.07 indicates acceptable fit [46].

After the dimensions were identified, we calculated the corrected item-total correlation (CITC), which is the correlation between the item score and the total score without the item (i.e. restscore) [47]. The CITC needs to be calculated for each subscales, since items can only be summed into a score when they measure the same construct [48]. The CITC evaluates the degree to each item is coherent with the other items from the same subscale [49]. Given the ordinal nature of the data, the CITCs were calculated using non-parametric rank correlation Kendall’s τ [50] with bootstrapped CIs [51]. Items with CITCs higher than 0.30 were considered to be coherent with the subscale [52]. The Reliability was calculated with the Categorical Omega [53]. The advantage of the Omega coefficient is that it does not rely on restrictive assumptions of tau-equivalence as do other traditional coefficients, such as Cronbach’s α [54]. The analysis was conducted with R package *lavaan* [55]. All subsequent analysis, including the evaluation of the functioning of individual items (i.e. item analysis) and criterion validity, were also conducted on the validation sample.

#### Item analysis

After dimensionality and overall fit were established, we employed *item-response theory* to evaluate the EQ-5D-5L performance at an item level. We followed previous recommendations of the Rasch model (specifically, the Partial Credit Model for polytomous items) as a model of choice [14, 56, 57]. For example, the EuroQol Group employed the Partial Credit Model in the EQ-5D-5L initial validations [58], been later followed by other empirical research [59, 60]. In our study, the Rasch model (RM) for polytomous items, the Partial Credit model [61], was estimated with conditional maximum likelihood [62] and person parameters were estimated with weighted maximum likelihood (WML) [63]. As a means of sensitivity analysis, we also evaluated the Rating Scale model [64]. The Rating Scale model is a restricted version of the Partial Credit model which constrains the distance between item thresholds to be the same across all items. While the Rating Scale model is more parsimonious, the assumption of equal threshold distance across all items can be restrictive and incompatible with certain questionnaires in health sciences (see, for instance, Shea, Tennant [65]). Hence, a Likelihood Ratio Test (LRT) was conducted to determine whether the Partial Credit model or the Rating Scale model better explained the EQ-5D-5L item responses. The LRT null hypothesis is that differences between the restricted model, the Rating Scale model, and the Partial Credit model occur only due to sampling variation. Thus, a significant LRT indicates that Partial Credit model fitted the data better than the Rating Scale model [65].

Once the Partial Credit model or Rating Scale model were selected, fit to the RM was evaluated with the Conditional Likelihood Ratio (CLR) test [66]. Item fit was evaluated with *conditional* infit and outfit statistics [67] with bootstrapped standard errors [68]. We report item discrimination and threshold parameters. In the Partial Credit model and Rating Scale model, discrimination parameters are constrained to 1. The thresholds parameters indicate in the latent trait scale (i.e. HRQoL) the point of equal probability of choosing between two adjacent categories (e.g. “moderate” and “severe problems”) [69]. In addition, Item Characteristic Curves (ICCs) were plotted for a graphical inspection of item fit. Ideally, ICCs would display average observed item responses for each possible total score. However, since it is unlikely that the sample will contain a meaningful number of respondents for each possible total score, we created 5 class intervals [70].

Finally, we evaluated the adequacy of the EQ-5D-5L five categories through the visual inspection of Category Characteristic Curves. In polytomous items, it is expected that increasing amounts of the latent trait (i.e. poor health-related quality of life) will correspond to a monotonically increasing endorsement of response categories associated with poor HRQoL. For example, it is expected that respondents with poor HRQoL will have a higher probability of endorsing a category such as “I have extreme pain or discomfort” rather than “I have slight pain or discomfort”. Moreover, in case all EQ-5D-5L five categories are necessary to evaluate health-related quality of life, it is expected that each category will become the most probable for at least a certain range of HRQoL [71]. The analysis was conducted with DIGRAM v4.03 [72] and R package *iarm* [73].

#### Criterion validity

To evaluate concurrent validity, we initially followed a ‘risk factor’ approach [22] and used dichotomized outcomes (e.g. good/fair health and poor health) to investigate whether the EQ-5D-5L scores could discriminate between individuals with high risk and low risk of poor general health, chronic pain and experiences of racial discrimination. To do so, we investigated the AUROC between EQ-5D-5L item scores (and subscale scores) and measures of general health and chronic pain. The AUROC indicates the probability that a randomly chosen participant with poor general health is correctly identified by having poor HRQoL (measured by EQ-5D-5L scores) compared to a randomly chosen participant with good/fair HRQoL. Similarly, the AUROC indicates the probability that a randomly chosen participant experiencing chronic pain is correctly identified by having poor HRQoL compared to a randomly chosen participant with no chronic pain. Thus, it was expected AUROC values higher than 50%, indicating that EQ-5D-5L scores identified participants with poor general health and chronic pain with a higher probability than random chance (50%).

We also inspected the AUROC between EQ-5D-5L item scores (and subscales scores) and the score derived for experiences of racial discrimination to evaluate discriminant validity. It is theoretically expected that, besides the domain of anxiety/depression, there is weak or no association between mobility, self-care, usual activities or pain/discomfort with experiences of racial discrimination. Thus, expected AUROC values should be closer to 50%, indicating that EQ-5D-5L scores were not able to identify participants who experienced more frequent episodes of racial discrimination better than chance alone.

Since dichotomous variable can lead to loss of information under certain circumstances [74], to evaluate the robustness of our findings, we also employed univariate linear regressions to evaluate the effect of the EQ-5D-5L individual items and the participants’ latent trait scores (i.e. person parameters) on the original variables of general health (i.e. 5-point measure of general health) and experiences of racism (i.e. MIRE total score) before dichotomisation. Since the 5-point measure of general health (1 = Excellent, 2 = Very good, 3 = Good, 4 = Fair, 5 = Poor) and MIRE total score (ranging from 9 to 45) are on different scales, we report standardized regression coefficients.

## Results

The participants' characteristics are displayed in Table 1. The majority of participants had education up to finishing high school (67.4%), were unemployed or on benefits (74.9%) and did not have access to a health care card (75.4%). The average age was 39.7 years (Median = 37 years) and approximately 45 percent of the sample was aged 40 years or older. Two-thirds of the participants were female and more than 60 percent resided in non-metropolitan locations.

**Table 1:** Characteristics of study participants

	HPOVC Study (n = 988)	
	n	%
Age		
Mean (SD)	39.7 (15.1)	
Missing	7	0.7
Sex		
Male	334	33.8
Female	654	66.2
Missing	0	0.0
Location		
Regional	617	62.5
Metropolitan	369	37.3
Missing	2	0.2
Education		
Up to High School	666	67.4
Technical education or university	312	31.6
Missing	10	1.0
Employment		
Employed	239	24.2
Unmployed/Benefits	740	74.9
Missing	9	0.9
Access to health care card		
Yes	202	20.4
No	745	75.4
Don’t know/Missing	41	4.2

Mean values, minimum, maximum and standard deviations; numbers and percentages

### Dimensionality

**Fig. 1:**
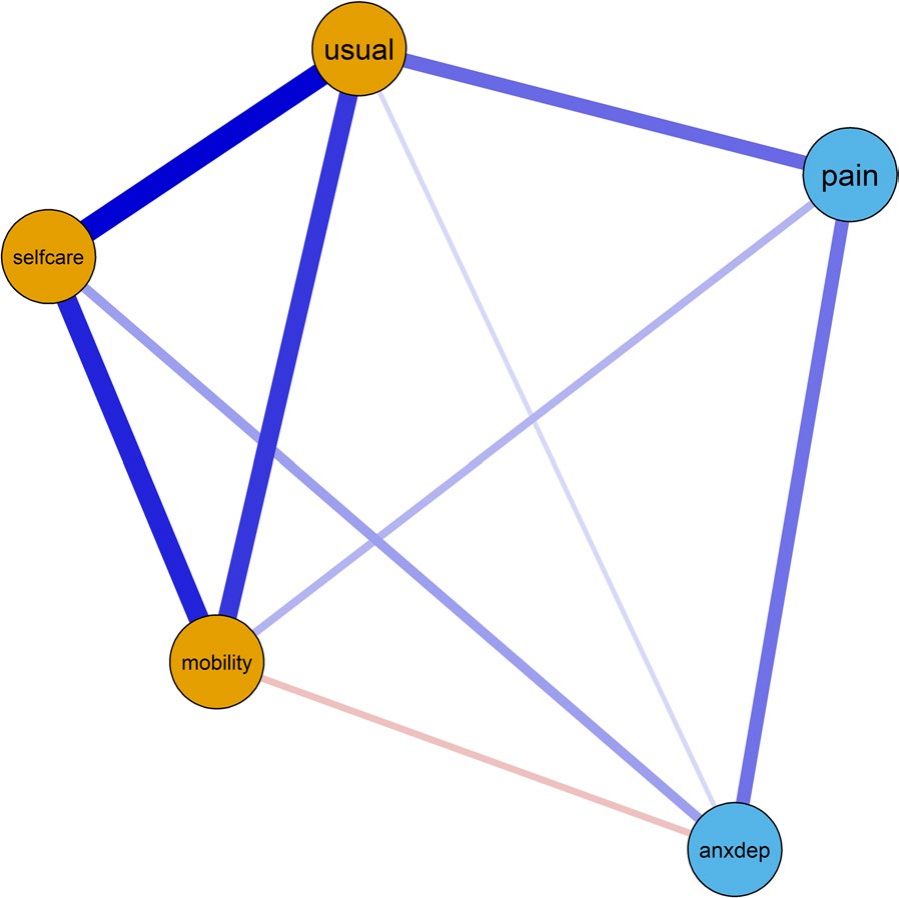
Network of the EQ-5D-5L. Note: Nodes represent items and edges represent partial correlation coefficients. The orange nodes indicate the EGA identified “Physiological” dimension, while the blue nodes indicate the EGA identified “Psychological” dimension. Positive edges are plotted as blue lines and negative edges are plotted as red lines. The thickness and saturation of edges indicate the strength of the regularised partial correlations

The EGA indicated that the EQ-5D-5L has a two-dimensional structure. The first dimension, comprising items “Mobility”, “Self-care” and “Usual activities”, was named the “Physiological” dimension. The second dimension, comprising the items, “Anxiety/Depression” and “Pain”, was named the “Psychological” dimension. The network representation of the EQ-5D-5L is displayed in Fig. 1.

The network edges (i.e. regularised partial correlations between items) are displayed in Table 2.

**Table 2:** Network edges of the EQ-5D-5L

	Mobility	Self-care	Usual activities	Pain/Discomfort	Anxiety/Depression
Mobility	1.00				
Self-care	0.27	1.00			
Usual activities	0.42	0.53	1.00		
Pain/Discomfort	0.20	0.00	0.16	1.00	
Anxiety/Depression	− 0.12	0.23	0.04	0.34	1.00

The network edges indicate regularised partial correlations between items. The matrix of network edges is symmetrical so values above the diagonal were omitted

The application of EGA to 2500 bootstrap samples showed that 2 dimensions were identified in 91.6% of the bootstrap samples, while 1 dimension was identified in 2.4% of the bootstrap samples and 5 dimensions were identified in 6.0% of the bootstrap samples. These results indicate that the 2-dimensional structure identified was stable across the bootstrap samples; that is, it is unlikely that the identification of the 2-dimensional structure by EGA was merely a consequence of sampling variation.

### Model fit

After the 2-dimensional structure was identified by EGA in the test sample, we compared it with the 1-dimensional structure in the validation sample to investigate which structure received more support from the data. Table 3 shows that the fit of the 1-dimensional structure was mixed since RMSEA (> 0.07) had unacceptable values. On the other hand, the fit of the 2-dimensional structure was excellent since both CFI (> 0.96) and RMSEA (< 0.07) achieved desirable values.

**Table 3:** Model fit comparison of the 1-dimensional structure and the 2-dimensional structure identified by EGA

Model	χ^2^	*df*	*p-*value	RMSEA	90% CI	CFI
*EQ-5D-5L*						
1-dimensional structure	55.2	5	< 0.001	0.143	[0.110, 0.178]	0.975
2-dimensional structure	10.3	4	0.04	0.057	[0.013, 0.100]	0.997

χ2 = scaled chi-square; df = degrees of freedom; RMSEA = scaled root mean square error of approximation; CFI = scaled comparative fit index

The CITCs between the items “Mobility” (CITC = 0.87—95% CI [0.84, 0.90]), “Self-care” (CITC = 0.53—95% CI [0.47, 0.59]) and “Usual activities” (CITC = 0.81—95% CI [0.77, 0.85]) with the “Physiological” subscale were moderate to strong. The CITCs between the items “Anxiety/Depression” (CITC = 0.77—95% CI [0.74, 0.80]) and “Pain/Discomfort” (CITC = 0.71—95% CI [0.68, 0.75]) with the “Psychological” subscale were strong. In both cases, the CITCs of all items were above suggested cut-off values (> 0.30) indicating that the “Physiological” and “Psychological” subscales are constituted by a cohesive set of items. Reliability of the Physiological scale was good (Ω_c_ = 0.84—95% CI [0.79, 0.89]), while reliability of the Psychological subscale was adequate (Ω_c_ = 0.70—95% CI [0.63, 0.74]).

### Item analysis

The Likelihood Ratio test (LRT) indicated that Partial Credit model was a significantly better fit to the data compared to the Rating Scale model for the Physiological subscale (χ2 (6) = 13.52, p = 0.03) but there was no significant difference between the fit of both models to the Psychological subscale (χ2 (3) = 1.42, p = 0.70). While there was no significant difference in the fit of the Partial Credit model and the Rating Scale model to the Psychological subscale, the Rating Scale model was not appropriate for the Physiological subscale. Hence, to avoid employing different measurement models with distinct assumptions about the item response process for the two EQ-5D-5L subscales (i.e. Rating Scale model for Psychological subscale and Partial Credit model for the Physiological subscale), the EQ-5D-5L item responses were evaluated with the measurement model that was adequate for *both* subscales, the Partial Credit model.

The Physiological subscale (χ^2^(11) = 2.55, p = 0.99) and the Psychological subscale (χ^2^(7) = 8.74, p = 0.27) achieved overall fit to the Partial Credit model. The fit to the Partial Credit model was also confirmed at the item level (Table 4). The observed infits and outfits were similar in magnitude (and not statistically different) from the expected value of 1 under the Partial Credit model. The CLR and observed infits and outfits further confirmed the adequacy of the Partial Credit model to model item responses to the Physiological and the Psychological subscales.

**Table 4:** Item fit statistics for the EQ-5D-5L

	Conditional Outfit	Conditional Infit		
Item	Observed	*p*-value	Observed	*p*-value
*Physiological subscale*				
Mobility	0.994	0.940	1.005	0.920
Self-care	0.927	0.620	0.917	0.490
Usual movements	1.026	0.640	1.034	0.490
*Psychological subscale*				
Pain/Discomfort	1.000	0.960	1.008	0.160
Anxiety/Depression	1.000	0.960	1.008	0.160

The Conditional Outfit and Conditional Infit statistics have expected values equal to one under the Rasch model

**Fig. 2:**
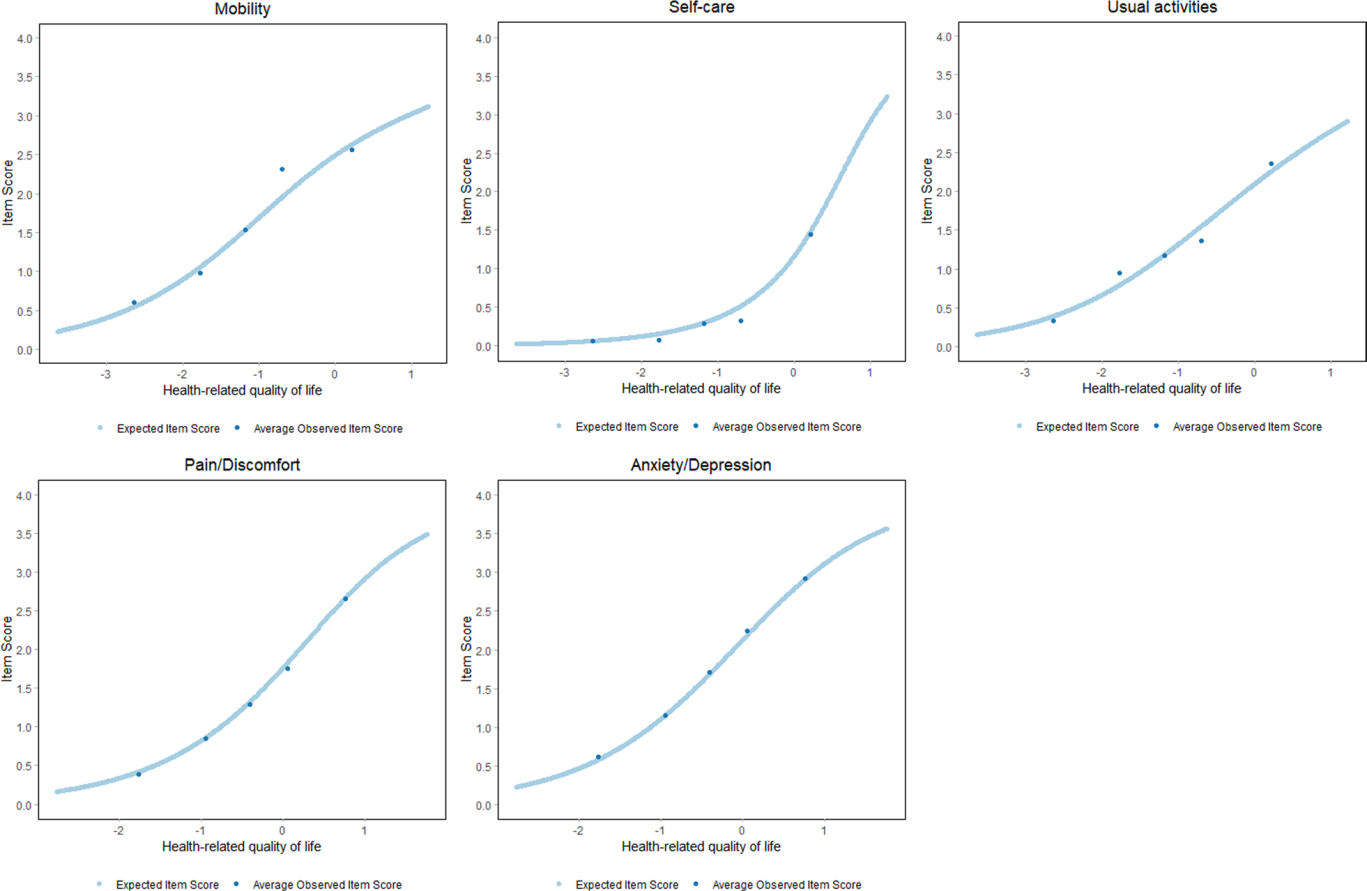
Item characteristic curves of the EQ-5D-5L items. Note: The x-axis displays HRQoL with higher values indicating worse HRQoL. The y-axis displayed EQ-5D-5L item scores. The dark blue points represent the average observed item responses in each class interval. The light blue logistic curve indicates the expected item responses under the Rasch model

The graphical inspection of the Item Characteristic Curves confirmed that average observed item responses were in accordance with the item responses expectations (Fig. 2). Additionally, the average observed item responses *in general* monotonically increased given the values of HRQoL. That is, respondents with poorer HRQoL increasingly endorsed EQ-5D-5L categories indicating more problems with mobility, self-care, usual activities, pain/discomfort and anxiety/depression.

The item discrimination and threshold parameters are reported in Table 5.

**Table 5:** Item parameters of the EQ-5D-5L

	Discrimination	Threshold 1	SE	Threshold 2	SE	Threshold 3	SE	Threshold 4	SE
*Physiological subscale*									
Mobility	1.00	− 2.49	0.30	− 1.10	0.28	− 0.02	0.31	1.98	
Self-care	1.00	0.08	0.27	**0.63**	0.44	**0.36**	0.58	1.06	0.71
Usual movement	1.00	− 2.15	0.28	− 0.38	0.28	0.53	0.38	1.50	0.63
*Psychological subscale*									
Pain/discomfort	1.00	− 0.80	0.15	0.00	0.16	0.37	0.23	0.96	0.36
Anxiety/depression	1.00	− 1.01	0.17	− 0.60	0.16	0.44	0.20	0.64	0.28

SE = standard error. The discrimination parameters do not have standard error since these parameters were constrained to 1 under the Partial Credit Model. Item disordered thresholds are highlighted in bold

**Fig. 3:**
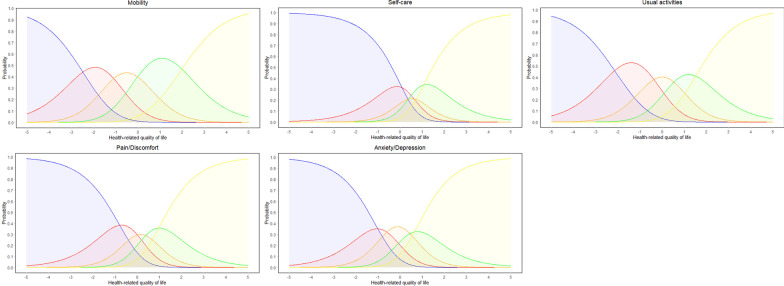
Category Characteristic Curves of the EQ-5D-5L items. Note: The blue category was “I have no problems/I am not”, the red category was “I have slight problems/I am slightly”, the orange category was “I have moderate problems/I am moderately”, the green category was “I have severe problems/I am severely” and the yellow category was “I have extreme problems/I am extremely”

The investigation of Category Characteristic Curves (Fig. 3) showed that the categories of all EQ-5D-5L items were correctly ordered and became the most probable for a specific range of HRQoL. The only exception was the Self-Care item which had disordered thresholds (Table 5), so the middle category (“I have moderate problems washing or dressing myself”) never became the most probable category for the EQ-5D-5L respondents.

### Criterion validity

**Fig. 4:**
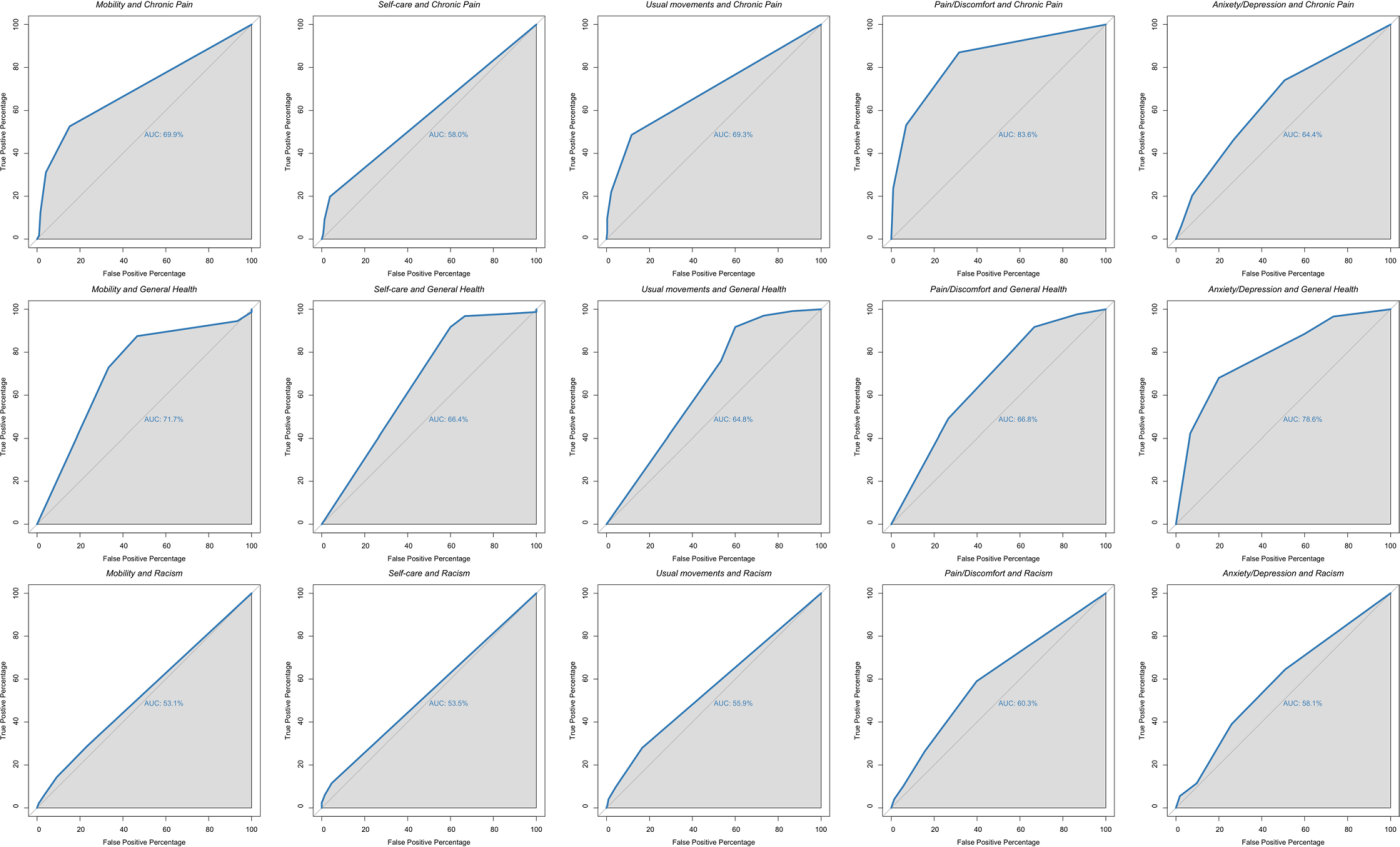
Area under the receiver operating characteristic (AUROC) curves for the five EQ-5D-5L items predicting chronic pain, general health and experiences of discrimination. Note: The x-axis displays “False Positive Percentage”, while the y-axis displays “True Positive Percentage”. The first row indicates AUROC for chronic pain (expected AUROC > 50%). The second row indicates AUROC for general health (expected AUROC > 50%). The third row indicates AUROC for experiences of discrimination (expected AUROC ~ 50% for mobility, self-care, usual activities or pain/discomfort with experiences of racial discrimination; expected AUROC > 50% for anxiety/ depression)

The AUROC values substantially above 50% indicated that EQ-5D-5L individual items were able to correctly identify participants with poor general health and chronic pain (Fig. 4). Figure 4 shows, for example, that the probability of identifying a participant experiencing chronic pain through high scores on the “Pain/Discomfort” item was 83.6% higher than if the participant was not experiencing chronic pain (first row, fourth column). These findings suggest good concurrent validity of the EQ-5D-5L five individual items.

Furthermore, when experiences of racial discrimination were considered, AUROC values of items from the “Physiological” subscale, such as mobility (AUROC = 53.1%), self-care (AUROC = 53.5%) and usual activities (AUROC = 55.9%), were close to 50%. That is, according to the expectations, scores of these three items had a weak association with experiences of racial discrimination and were able to identify participants that experienced racial discrimination just slightly better than random chance. The same was observed regarding the items from the “Psychological” subscale, such as anxiety/depression (AUROC = 58.1%) and pain/discomfort (AUROC = 60.3%), which were also poor predictors of racial discrimination. These results indicate that the EQ-5D-5L displayed reasonable discriminant validity.

**Fig. 5:**
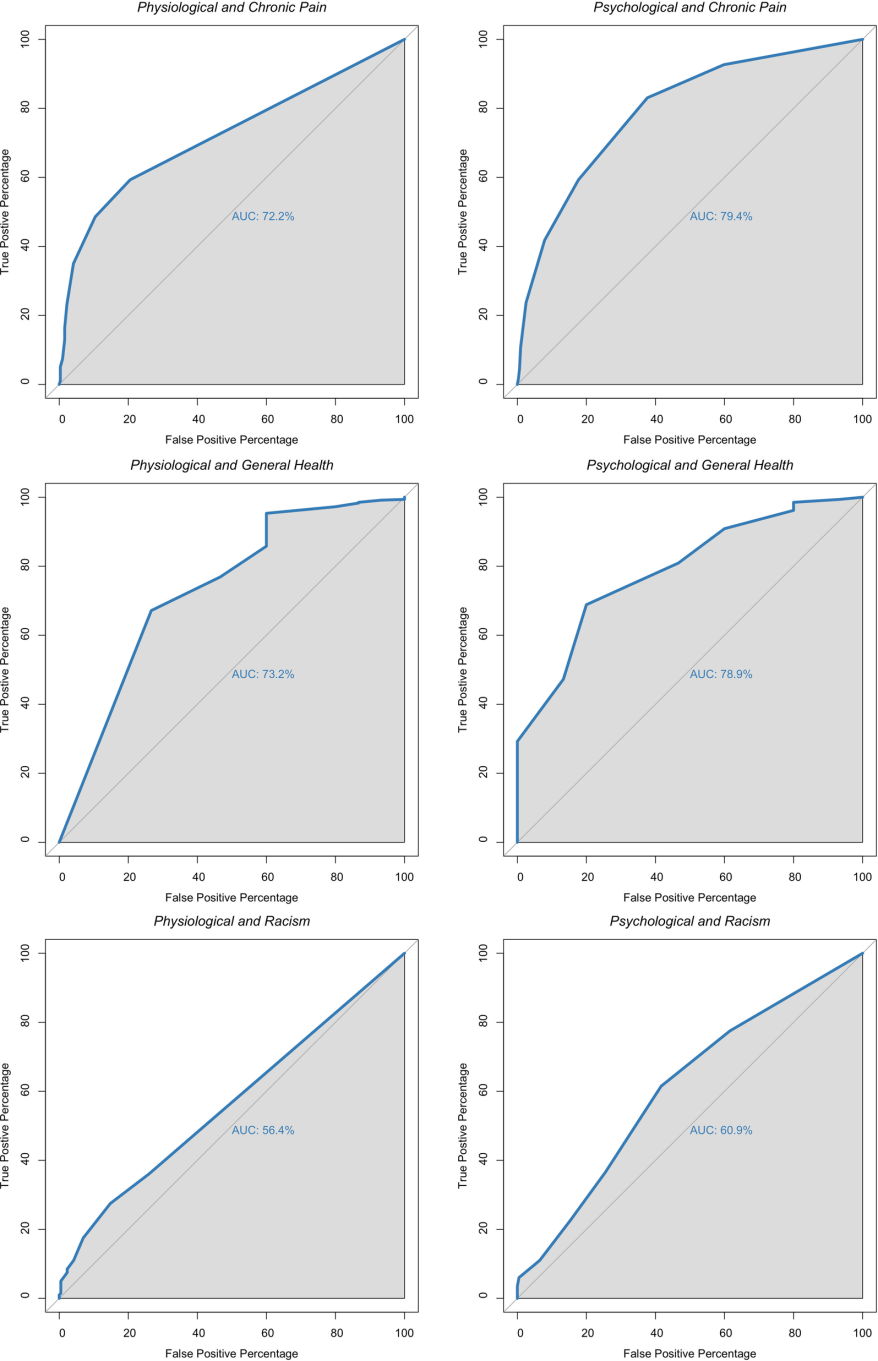
Area under the receiver operating characteristic (AUROC) curves for the two EQ-5D-5L “Physiological” and “Psychological” dimensions predicting chronic pain, general health and experiences of discrimination. Note: The first row indicates AUROC for chronic pain. The second row indicates AUROC for general health. The third row indicates AUROC for experiences of discrimination

The concurrent and discriminant validity and the AUROCs of the two identified dimensions, Physiological and Psychological subscales, are displayed in Fig. 5.

Figure 5 indicates that the dimensions of Physiological and Psychological subscales also displayed good concurrent and discriminant validity. For instance, high scores on the Physiological subscale score were able to identify more than 70% of the time participants experiencing chronic pain (AUROC = 72.2%) or poor general health (AUROC = 73.2%) compared to participants who were not. Moreover, when the Psychological subscale scores were used, these numbers increased to almost 80% for both chronic pain (AUROC = 79.4%) and poor general health (AUROC = 78.9%). Regarding discriminant validity, the Physiological subscale scores were able to identify participants who experienced racism 56% of the time (AUROC = 56.4%) and the Psychological subscale scores identified participants who experienced racism 61% of the time (AUROC = 60.9%), only marginally better than random chance. Both scales were poor predictors of individuals who experienced racism. These results indicate that the EQ-5D-5L good concurrent and discriminant validity not only on an item level but also on a dimension/subscale level.

Finally, the results were also consistent when the non-dichotomous variables were used, the 5-point measure of general health (1 = Excellent, 2 = Very good, 3 = Good, 4 = Fair, 5 = Poor) and MIRE total score (ranging from 9 to 45), were used. For instance, the effects of mobility ($\beta$=0.31—95% CI [0.22, 0.39]), self-care ($\beta$=0.20—95% CI [0.12, 0.29]), usual activities ($\beta$=0.34—95% CI [0.25, 0.42]), pain/discomfort ($\beta$=0.33—95% CI [0.24, 0.41]) and anxiety/depression ($\beta$=0.29—95% CI [0.21, 0.38]) on general health were stronger than the effects of mobility ($\beta$=0.13—95% CI [0.03, 0.24]), self-care ($\beta$=0.10—95% CI [− 0.01, 0.20]), usual activities ($\beta$=0.12—95% CI [0.01, 0.23]), pain/discomfort ($\beta$=0.17—95% CI [0.07, 0.27]) and anxiety/depression ($\beta$=0.14—95% CI [0.04, 0.23]) on experiences of racism. Moreover, when evaluated at a dimension/subscale level, the effects of Physiological ($\beta$=0.36—95% CI [0.27, 0.44]) and Psychological ($\beta$=0.37—95% CI [0.29, 0.46]) latent trait scores on general health were also stronger than the effects of Physiological ($\beta$=0.14—95% CI [0.04, 0.25]) and Psychological ($\beta$=0.20—95% CI [0.10, 0.29]) latent trait scores on experiences of racism. The results further confirm the association between EQ-5D-5L items and subscales with general health indicating convergent validity, while the weak association between EQ-5D-5L items and subscale and experiences of racism support discriminant validity.

## Discussion

This study aimed to evaluate the construct validity and reliability of the EQ-5D-5L for an Aboriginal Australian population. We employed a multi-method approach to comprehensively evaluate the EQ-5D-5L psychometric properties in a large sample, both at an instrument level and at an item level. We also investigated whether EQ-5D-5L scores could correctly identify participants with poor general health and chronic pain. To the best of our knowledge, this is the first study to evaluate the EQ-5D-5L psychometric properties in any Indigenous population [16].

Our findings showed that EQ-5D-5L psychometric properties were excellent. The instrument is composed of two dimensions, Physiological and Psychological, and reliability was adequate. Moreover, the EQ-5D-5L provides a health-state classification system that is amenable to future valuation using preference-based techniques. Future research should also investigate whether the instrument can potentially be expanded to also incorporate other domains specific of Aboriginal Australians’ HRQoL, such as cultural health, knowledge and interaction with the health system, among others [75]. Implications for practice are provided.

### Dimensionality

The findings indicated two overall dimensions, Physiological and Psychological, in an Aboriginal population. The distinction between a “physiological” and a “psychological” dimension is theoretically consistent with the current understanding about Aboriginal and Torres Strait Islanders’ SEWB. For instance, the nine domains that typically characterise Aboriginal SEWB include “physical health” and “mental health” [6]. These two domains have also been previously referred as “Connection to the body” and “Connection to mind and emotions”, respectively [76]. Finally, in a recent qualitative study, Aboriginal parents described the importance of the “physical” and “emotional” domains in the HRQoL of their children [75].

In other Indigenous groups, previous validations of HRQoL instruments also identified similar “physiological” and “psychological” dimensions. For example, in Native Americans, “Symptoms” and “Psychological Impact” (in addition to “Community and Social Restrictions”) were determined as dimensions of HRQoL [77]. In New Zealand, the distinction between “mental” and “physical” dimensions was also identified in Maori people, although older Maori (more than 45 years old) did not make the same differentiation. These results led Scott, Sarfati [78] to suggest that younger Maori perceived their HRQoL more similar to Western cultures, in which HRQoL questionnaires “measure largely independent dimensions of physical and mental health”.

In summary, our findings indicated that the EQ-5D-5L was comprised by two distinct dimensions, “physiological” and “psychological”. While these dimensions were consistent with theoretical understanding regarding Aboriginal and Torres Strait Islanders’ SEWB, there are two important things to be noticed. Firstly, it is unlike that the five items measuring mobility, self-care, usual activities, pain/discomfort and anxiety/depression are enough to *exhaust* the “physiological” and “psychological” dimensions of Aboriginal HRQoL. That is, there are also other factors that also potentially constitute the “physiological” and “psychological” dimensions of Aboriginal health. Secondly, “physiological” and “psychological” were the only two dimensions measured by the EQ-5D-5L. The EQ-5D-5L did not encompass other important dimensions of Aboriginal HRQoL. One of these dimensions, for example, is “cultural health”, which includes Aboriginal values, historical perspective in Australia and connection to the land [75]. We provide an in-depth discussion of this issue in the section “Limitations and future directions” and directions for future research.

### Item performance

Our findings showed robust evidence of the EQ-5D-5L validity at an item level. For instance, the fit of the items to the Rasch Model entails excellent measurement properties [79]. For our intended purposes, two important properties displayed by the EQ-5D-5L items were *monotonicity* and *adequacy of response categories*. Regarding monotonicity, the results showed that, on average, respondents with increasingly worse HRQoL monotonically endorsed higher scores on individual EQ-5D-5L items. Herein, we can be confident that higher scores on EQ-5D-5L items are correctly measuring higher values of the underlying construct (in this case, higher scores indicate worse HRQoL).

Moreover, the development of the EQ-5D-5L by the EuroQol Group (which has five response categories) occurred due to limited sensitivity of the EQ-5D-3L (which has three responses categories) to detect changes in health, in part due to ceiling and floor effects [58]. Regarding the adequacy of response categories, our findings showed that all five categories (from “I have no problems/I am not” to “I have extreme problems/I am extremely”) were the most probable category of choice for a specific group of respondents according to their level of HRQoL. For example, patients with moderate HRQoL had a higher probability of endorsing the category “I have moderate problems/I am moderately”, while participants with very poor HRQoL were more likely to endorse the “I have extreme problems/I am extremely” category.

These findings are in accordance with previous research that the inclusion of five categories in the EQ-5D-5L was an improvement upon the EQ-5D-3L measurement properties in multiple countries [17, 80]. In our study, the only exception was the Self-Care item middle category (“I have moderate problems washing or dressing myself”), which never became the most probable category of choice for the Aboriginal respondents. Before changes in the instrument are made (such as collapsing categories), future independent studies with other Aboriginal populations should investigate whether problems with this category will re-appear. In summary, our investigation of the EQ-5D-5L five categories provided evidence that “category definitions are adequate (not too narrow in definition) and that responders have not been presented with overwhelming category options” [81].

### Implications for practice

The identification of two dimensions ensue implications for future use of the EQ-5D-5L in Aboriginal Australian populations. Our findings support that the EQ-5D-5L can be used as five independent items *or* as two broader “Physiological” and “Psychological” dimensions.

Firstly, the five EQ-5D-5L individual items showed good discriminatory power to identify respondents with poor self-rated general health and chronic pain. While fit to the Rasch Model indicated equal item discrimination regarding the latent trait (Physiological and Psychological), items can display different discrimination regarding outcomes, such as criterion variables [82]. The items’ discriminatory power was mostly consistent with theoretical expectations. For example, the EQ-5D-5L item which better-discriminated participants who experienced significant pain over the last 6 months (or more) was the item “Pain/Discomfort”, while the second item with highest discriminatory power was “Mobility”. These findings provided support for the use of the EQ-5D-5L items as stand-alone items, which is the most common EQ-5D-5L usage [12]. Secondly, in case researchers are interested in the computation of total scores to evaluate the EQ-5D-5L at a *domain* level, the investigation of dimensionality showed that two subscales scores should be computed, one for the “Physiological” and one for the “Psychological” dimension (instead of one total score summing all five items).

### Limitations and future directions

The SEWB of Indigenous Australians is a multidimensional and multifaceted construct. The holistic nature of SEWB, which includes several dimensions such as “family and community”, “autonomy, empowerment and recognition”, “work, roles and responsibilities”, and “education” (Butler 2019), indicates that the health (and, consequently, HRQoL) of Aboriginal Australian populations is conceptualized differently to Western societies [83, 84]. Hence, many Western-developed HRQoL instruments (including the EQ-5L-5D) that are built upon more narrow conceptions of health are likely to overlook many aspects of health and wellbeing that are valued by Indigenous people [16].

In our study, in the initial consultation prior to the instrument application, the Indigenous Reference Group recommended the EQ-5D-5L as a potentially valid instrument to capture specific aspects of HRQoL until a broader instrument is available. Moreover, they also required that further evaluation should be conducted to investigate the EQ-5D-5L psychometric properties. Our findings showed that EQ-5D-5L items provided correct measurement of five aspects (mobility, self-Care, usual activities, pain/discomfort, anxiety/depression) of Aboriginal HRQoL and that these five aspects clustered into two overall dimensions (“Physiological” and “Psychological” dimensions). While the EQ-5D-5L was found to be an appropriate instrument to measure these specific aspects of Aboriginal HRQoL, the EQ-5D-5L limitation in scope must always be considered. For instance, the EQ-5D-5L items do not exhaust Aboriginal Australians dimensions of “Physiological” and “Psychological” health or cover other relevant dimensions, such as “family and community” and “autonomy, empowerment and recognition”.

Directions for future research include potentially expanding the EQ-5D-5L to encompass these other domains less common in Western conceptualizations of HRQoL [16] and, consequently, lead to an expanded instrument specific to Aboriginal Australian populations. These studies can implement, for instance, focus groups to investigate the relevant domains and develop new culturally-specific items. Then, these items can be piloted in a population and the psychometric properties of the expanded instrument studied [85].

## Conclusions

In this study, we employed a multi-method approach to comprehensively evaluate the psychometric properties of the EQ-5D-5L in a large sample of Aboriginal Australians in South Australia. The evidence showed that the EQ-5D-5L displayed excellent psychometric properties and is a potentially valid instrument to measure five specific aspects (Mobility, Self-Care, Usual activities, Pain/Discomfort, Anxiety/Depression) of Aboriginal and Torres Strait Islander HRQoL. Moreover, the EQ-5D-5L provides a health-state classification system that is amenable to future valuation using preference-based techniques. A future research agenda comprises the investigation of other domains of Aboriginal and Torres Strait Islander HRQoL and potential expansions to the instrument.

## Data Availability

The datasets generated and/or analysed during the current study are not publicly available since we do not have permission from the ethics committee to publicly release the datasets in either identifiable or de-identified form. The datasets are available from the corresponding author on reasonable request.

## Abbreviations

AUROC: area under the receiver operating characteristic curve
CITC: corrected item-total correlation
CFA: confirmatory factor analysis
CLR: conditional likelihood ratio
EBIC: extended Bayesian information criteria
EGA: exploratory graph analysis
GGM: Gaussian graphical model
HPV: human papillomavirus
HRQoL: health-related quality of life
LASSO: least absolute shrinkage and selection operator
LRT: likelihood ratio test
MIRE: measure of indigenous racism experiences
NACCHO: national aboriginal community controlled health organisation
SEWB: social and emotional wellbeing
QALYs: quality-adjusted life years
WML: weighted maximum likelihood
